# Crossover shortage in potato is caused by *StMSH4* mutant alleles and leads to either highly uniform unreduced pollen or sterility

**DOI:** 10.1093/genetics/iyad194

**Published:** 2023-11-07

**Authors:** Corentin R Clot, Dennis Klein, Joey Koopman, Cees Schuit, Christel J M Engelen, Ronald C B Hutten, Matthijs Brouwer, Richard G F Visser, Martina Jurani, Herman J van Eck

**Affiliations:** Plant Breeding, Wageningen University & Research, Wageningen, 6700 AJ, The Netherlands; Graduate School Experimental Plant Sciences, Wageningen University & Research, Wageningen, 6708 PB, The Netherlands; Plant Breeding, Wageningen University & Research, Wageningen, 6700 AJ, The Netherlands; Plant Breeding, Wageningen University & Research, Wageningen, 6700 AJ, The Netherlands; Bejo Zaden B.V., Warmenhuizen, 1749 CZ, The Netherlands; Plant Breeding, Wageningen University & Research, Wageningen, 6700 AJ, The Netherlands; Plant Breeding, Wageningen University & Research, Wageningen, 6700 AJ, The Netherlands; Plant Breeding, Wageningen University & Research, Wageningen, 6700 AJ, The Netherlands; Plant Breeding, Wageningen University & Research, Wageningen, 6700 AJ, The Netherlands; Plant Breeding, Wageningen University & Research, Wageningen, 6700 AJ, The Netherlands; Plant Breeding, Wageningen University & Research, Wageningen, 6700 AJ, The Netherlands

**Keywords:** meiosis, crossover shortage, unreduced gametes, potato breeding, fertility, *StMSH4*, Plant Genetics and Genomics

## Abstract

The balanced segregation of homologous chromosomes during meiosis is essential for fertility and is mediated by crossovers (COs). A strong reduction of CO number leads to the unpairing of homologous chromosomes after the withdrawal of the synaptonemal complex. This results in the random segregation of univalents during meiosis I and ultimately to the production of unbalanced and sterile gametes. However, if CO shortage is combined with another meiotic alteration that restitutes the first meiotic division, then uniform and balanced unreduced male gametes, essentially composed of nonrecombinant homologs, are produced. This mitosis-like division is of interest to breeders because it transmits most of the parental heterozygosity to the gametes. In potato, CO shortage, a recessive trait previously referred to as desynapsis, was tentatively mapped to chromosome *8*. In this article, we have fine-mapped the position of the CO shortage locus and identified *StMSH4*, an essential component of the class I CO pathway, as the most likely candidate gene. A 7 base-pair insertion in the second exon of *StMSH4* was found to be associated with CO shortage in our mapping population. We also identified a second allele with a 3,820 base-pair insertion and confirmed that both alleles cannot complement each other. Such nonfunctional alleles appear to be common in potato cultivars. More than half of the varieties we tested are carriers of mutational load at the *StMSH4* locus. With this new information, breeders can choose to remove alleles associated with CO shortage from their germplasm to improve fertility or to use them to produce highly uniform unreduced male gametes in alternative breeding schemes.

## Introduction

Meiosis is a specialized type of cellular division, essential for sexually reproducing organisms, which generates 4 haploid spores out of a single diploid mother cell. This is achieved via 2 successive divisions, meiosis I and II, following a single S-phase. While meiosis II resembles a haploid mitosis where sister chromatids are separated, meiosis I is characterized by pairing and subsequent separation of homologous chromosomes. During the prophase of meiosis I, homologous chromosomes pair and associate with each other via the synaptonemal complex in a process known as synapsis. Following withdrawal of the synaptonemal complex, the connexion between homologs is maintained until anaphase I by crossovers (COs). Those COs ensure the proper positioning and segregation of homologs during the first meiotic division by providing a counterforce to the pole-directed spindle forces (Page and Hawley 2003). A drastic reduction in CO numbers leads to the unpairing of most homologs at the end of prophase I, in a phenotype previously referred to as desynapsis (Giraldez and Lacadena 1976; Jongedijk and Ramanna 1988; Cai and Makaroff 2001). In clones with CO shortage, the resulting univalents will segregate randomly during meiosis I, ultimately producing aneuploid nonviable gametes. Such CO shortage has been observed in organisms defective in the class I CO pathway (Lynn *et al.* 2007; Pyatnitskaya *et al.* 2019). This pathway, also known as ZMM pathway (an acronym for Zip1-4, Msh4-5, and Mer3), is responsible for 75–85% of COs in *Arabidopsis thaliana* (Serrentino and Borde 2012). In addition to their role in chromosome segregation, COs are necessary for recombination events which reshuffle parental chromosomes into unique genetic combinations (Mercier *et al.* 2015). Although recombination and segregation are essential for genetic diversity, they can pose difficulties for breeders of heterozygous outcrossing crops, as it makes it challenging to preserve previously chosen combinations of beneficial alleles. The highly heterozygous autotetraploid potato (*Solanum tuberosum*) with its slow increase of genetic gains is one embodiment of such a crop. To circumvent this challenge, the potato breeding community is currently putting a lot of efforts in the conversion of the allogamous tetraploid germplasm into a diploid self-compatible germplasm. This new germplasm, compatible with inbreeding, should enable breeding strategies that preserve cumulative genetic gains and delivers true potato seeds (TPS) F1 varieties (Lindhout *et al.* 2011; Jansky *et al.* 2016; Zhang *et al.* 2021; de Vries *et al.* 2023). Alternatively, if recombination is suppressed, as in the case of asynaptic *StDMC1* RNAi mutants (Kumar *et al.* 2023), the genetic makeup of the parents can be fixed and potentially transmitted to their offspring. This could make it possible to produce TPS without the need for extensive inbreeding. This concept was first explored about 3 decades ago using potato clones that lacked COs and also produce unreduced gametes (Hermsen 1980; Jongedijk 1991). Monogenic recessive CO shortage mutants were identified in dihaploids extracted from the tetraploid variety Chippewa and in *S. tuberosum* Group Tuberosum and Phureja hybrids. Those mutations were proven to be allelic and unified under the locus name *Ds-1* (Jongedijk and Ramanna 1988). Locus *Ds-1* was tentatively mapped to chromosome *8* in the biparental diploid population CE (Jacobs *et al.* 1995). In this population, CO shortage mutants were characterized by a 90% reduction in CO number affecting equally male and female meiosis and resulting in the formation of ∼1 bivalent per meiocytes (Jongedijk and Ramanna 1989). Assuming that during meiosis I each univalent has an equal chance of moving to either pole, the probability to obtain a gamete with a normal chromosomal set up is $\frac{1}{2^{n-b}}$ with *n* being the haploid number of chromosomes and *b* being the number of remaining bivalents. For a diploid potato clone with CO shortage, we can estimate that a single reduced gamete out of over 2,000 will be balanced, which leads to sterility. However, fertility can be rescued by a second meiotic alteration producing highly uniform unreduced gametes by first division restitution (FDR) (Ramanna 1983). Those unreduced gametes, also known as 2n gametes (2nG), are formed when the outcome of meiosis I is restituted by the misorientation of meiosis II spindle such as in *ps1* and *jason A. thaliana* mutants (d’Erfurth *et al.* 2008; Erilova *et al.* 2009; De Storme and Geelen 2011). Whether the first division was balanced or chaotic has no impact, and meiosis II can progress with the equational division of the entire chromosomal complement. This phenomenon was recently exploited to rescue male fertility in haploid *A. thaliana* (Aboobucker *et al.* 2023). FDR 2n pollen production is not rare in potato and has been reported in the parents of the population used to map *Ds-1* (Mok and Peloquin 1975; Ramanna 1979).

In the current study, we exploit the joint segregation of CO shortage and FDR 2n pollen in a diploid potato population to fine map the *Ds-1* locus on the short arm of chromosome *8*. We identified *StMSH4* as candidate gene and discovered a 7 bp insertion in the second exon of the allele associated with CO shortage. Mining the growing number of potato assemblies, we discovered another allele with a 3,820 bp insertion at the same position and confirmed that both alleles cannot complement each other. We subsequently found that nonfunctional *StMSH4* insertion alleles are prevalent in European cultivars. Finally, we discuss the opportunities and limitations offered by CO shortage in the context of potato breeding.

## Materials and methods

### Plant materials

The diploid mapping population CE-XW comprising 1,536 full-sibs descends from a cross between 2 heterozygous potato clones named C (USW5337.3) and E (77.2102.37) with mixed ancestry of *S. tuberosum* Group Tuberosum and Phureja and *Solanum vernei* (Supplementary Fig. 1). Notably, this particular cross is identical to the one employed by Jongedijk and Ramanna (1988, 1989) to describe the *Ds-1* locus associated with CO shortage. CE-XW seedlings were grown under standard greenhouse conditions at Unifarm (Wageningen University and Research) in 2020 (Clot *et al.* 2023), and a subset of 500 individuals were grown from tubers the following year. Tubers were planted on 2021 April 12 in 5-L pots and grown outdoors in a screen cage equipped with sprinkler irrigation. The diploid populations CRH and ERH, used for a complementation test, descend from crosses between the *S. tuberosum* diploid clone RH89–039-16, used as male parent and clones C and E used as female parents (Supplementary Fig. 1). Populations CRH and ERH were sown on 2022 March 29. For CRH and ERH populations, 100 seedlings were transplanted in 11 × 11-cm pots and grown in a greenhouse at ambient temperatures and under 16 h of light.

In previous studies, Mok and Peloquin (1975) and Ramanna (1979) investigated the meiosis of clones C and E, the progenitors of our mapping population. They documented the presence of both parallel and fused metaphase II plates, resulting in the production of FDR unreduced pollen. Likewise, clone RH89-039-16, the parent of our complementation test populations, was also shown to produce unreduced male gametes genetically equivalent to FDR (Park *et al.* 2007). Consequently, we anticipate the occurrence of FDR unreduced pollen in our mapping and complementation test populations. As elaborated in the subsequent section, we posit that this phenomenon will allow us to distinguish CO shortage from other forms of sterility. This distinction arises from the rescue of chromosome missegregation during meiosis I by the presence of FDR. This hypothesis is substantiated by the fact that Jongedijk and Ramanna (1988) successfully obtained tetraploid progenies by intercrossing tetraploid clones with diploid C × E descendants lacking COs and producing unreduced pollen.

### Phenotyping CO shortage via pollen microscopy

**Fig. 1.**
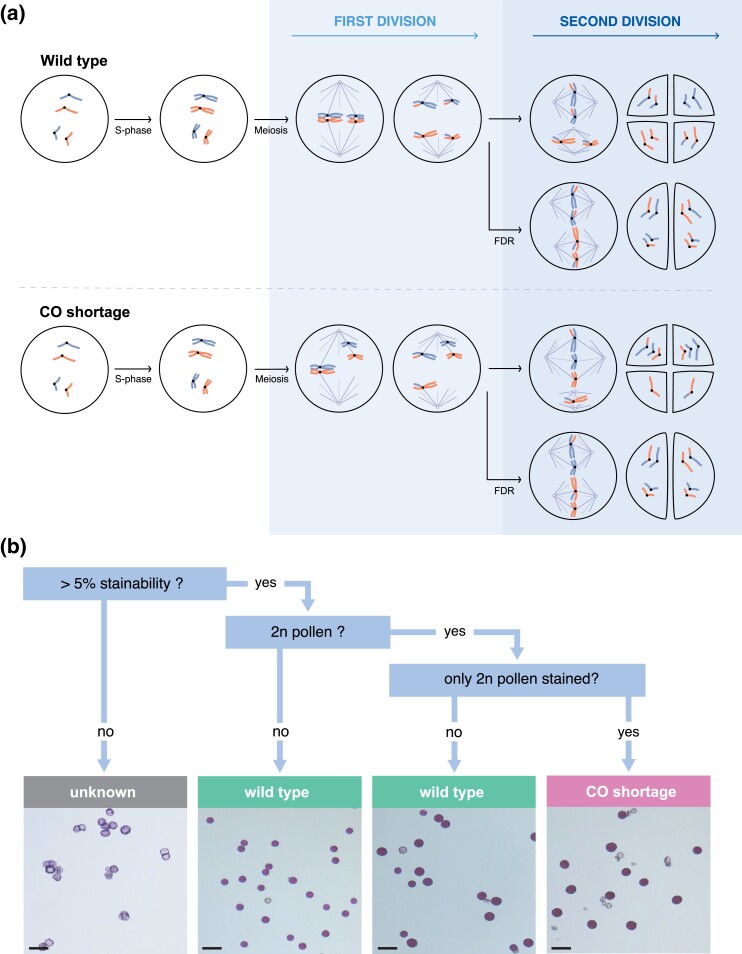
a) Schematic representation of wild-type and CO shortage mutant meiosis producing reduced and FDR 2n pollen. b) Dichotomous key used to phenotype CO shortage in population CE-XW via pollen microscopy. Scale bar is 50 µm.

During the flowering stage of the CE-XW population, which occurred between the 7th and the 10th week post-sowing in 2020 and between the 4th and 9th week post-planting in 2021, 1 pollen sample per flowering individual was collected. Pollen samples were extracted from a freshly opened flower at anthesis using a vibrator pin (modified electric toothbrush). Pollen was spread on a glass slide and stained with a simplified version of Alexander staining (Peterson *et al.* 2010) before being observed under bright field using a Axiophot Zeiss microscope equipped with a Neofluar ×10/0.30 lens. For each sample, 4 random field views containing ∼50 pollen grains each were used to visually estimate the proportion of stained pollen relative to the unstained or shriveled pollen grains. Within the fraction of stained pollen, the presence of 2n pollen, having a ∼15% larger diameter, was assessed. Combining those 2 observations, we trust that it is possible to identify individuals lacking COs. In a wild-type background, the effect of CO shortage on pollen stainability is undistinguishable from other forms of sterility. Therefore samples showing <5% of stained pollen grains were classified as unknown and excluded from the analysis. However, in the presence of another meiotic alteration resulting in incompletely penetrant FDR, the unbalanced chromosome segregation of the first meiotic division will be partially restored (Fig. 1a). We expect that this will result in the presence of stained balanced 2n pollen grains next to the shriveled, unstained, and unbalanced reduced pollen grains. The dichotomous key shown in Fig. 1b summarizes how we classified pollen samples into unknown, wild-type, and CO shortage classes based on pollen grain phenotypes. Samples showing a combination of large (2n) stained and small (n) unstained pollen grains were classified as CO shortage mutants. Conversely, samples showing more than 5% stainability and at least 1 small (n) and stained pollen grain were considered wild-type, irrespective of the level of 2n pollen production as we assumed that meiotic segregation occurred normally. When uncertain about either pollen stainability or pollen ploidy, the sample was classified as undetermined.

The same phenotyping protocol, with an increased stringency for wild-type classification set at more than 5% of small (n) stained pollen grains (instead of a single pollen grain), was applied to the CRH and ERH populations which flowered between the 9th and the 11th week post-sowing. In these populations, when in doubt about pollen size, pictures were taken with the Zeiss Axiocam ICc 5 color camera and pollen diameter was measured with ZEN 2.3 lite software. Pollen grains with a diameter above 23 µm were considered as 2n pollen.

### Cytological observations in male meiocytes

To confirm that our classification based on pollen microscopy corresponds to the expected cytological phenomenon of CO shortage and FDR, we observed male meiosis progression in clone CE-XW-1477, classified as CO shortage mutant, and in clone RH89-039-16 used as wild-type control. For both clones, floral buds of 3–4 mm were fixed in Carnoy solution [3:1 EtOH (99.8%): glacial acetic acid] which was refreshed once during the harvest day. Buds were kept overnight at 4°C before being washed twice with 70% EtOH and stored again at 4°C. Microscope slide were then prepared following the chromosome spread technique described by Jones and Heslop-Harrison (1996). After rinsing the fixated buds in water, we dissected the anthers and washed them a second time in water. The anthers were then incubated at 37°C for 1.2 h in a 1:1 mixture of pectolytic enzymes and 10 mM citrate buffer (pH 4.39). After enzymatic digestion, a single anther per slide was macerated in a small drop of 60% acetic acid and stirred gently on a hotplate at 55°C for 1 min. We then flooded the slide with freshly made Carnoy solution and dried it on a hotplate for 2 min. Finally, we stained the slides with 12 mL of DAPI diluted in VECTASHIELD (300 ng/µL) and mounted them with a coverslip. Slides were kept at 4°C until observed using a Axio Imager Z2 microscope equipped with an external light source (X-cite series 120 EXFO), a 120 W high-pressure metal halide lamp, a DAPI reflector, and an Axiocam 506 camera.

### Genetic analysis

Marker data and map construction of population CE-XW is detailed in Clot *et al.* (2023). A total of 4,894 biallelic female markers (encoded as h1 and h2 for haplotype 1 and 2) and 4,740 biallelic male markers (encoded as h3 and h4 for haplotype 3 and 4) segregating across 1,461 individuals were used. QTL mapping was performed using the package polyqtlR version 0.0.6 (Bourke *et al.* 2018). The function singleMarkerRegression was used to fit an additive model at each marker position returning the −log_10_  *P*-value of model fit per marker. The significance thresholds for QTL detection were determined via permutation tests on the phenotypic values with *N* = 1,000 cycles and α = 0.05. After an initial QTL discovery using separate maternal and paternal markers, QTL discovery was performed at full classification (h1h3, h1h4, h2h3, h2h4) obtained by merging parental markers of identical physical map position. This full classification was used for the recombinant analysis presented in Supplementary Table 1.

### Candidate gene exploration

To identify candidate genes for CO shortage, we used a gene and variant annotation approach. Firstly, we combined the high-confidence gene model annotation of DM v6.1 with their GO Slim annotations and descriptions based on their best hit with the *A. thaliana* proteome TAIR10. Secondly, we merged the bam files (see Clot *et al.* (2023) for details on alignment) of the 102 clones phenotyped as CO shortage mutant and genotyped with the allelic combination h1h3 at 1.15 Mb on chromosome *8* using SAMtools merge (Danecek *et al.* 2021). We then performed variant calling using BCFtools v.1.13 (Danecek *et al.* 2021) mpileup and call functions. Only variants which were also identified in either parents (see Clot *et al.* (2023)) with high confidence (%QUAL < 20 || FORMAT/DP > 40 || FORMAT/DP < 8) were kept for further analysis. We annotated the resulting high-confidence variants using SnpEff with default parameters and filtered them for loss-of-function (LOF) effect using SnpSift (Cingolani, Patel, *et al.* 2012; Cingolani, Platts, *et al.* 2012). We then parsed the DP4 field of the resulting VCF file to identify LOF mutations assumed to be fixed in the CO shortage bulk because neither forward no reverse read supported the reference allele (Supplementary Table 2). Finally, we retrieved the annotation of genes affected by homozygous LOF mutation to identify to most likely candidate gene.

### Retrieving haplotypes of the *StMSH4* locus

Haplotypes of *StMSH4* were mined from a collection of de novo–assembled potato genomes. A local blast database was build using nucleotide–nucleotide BLAST 2.8.1+ (Camacho *et al.* 2009) with a *Solanum lycopersicum* cv. ‘Heinz’ 170 build SL5.0 genome (Zhou *et al.* 2022) and 18 de novo–assembled potato genomes: M6 v.4.1 (Leisner *et al.* 2018); DM v.6.1 (Pham *et al.* 2020); Solyntus v.1.1 (van Lieshout *et al.* 2020); RH89-039-16 v.3 (Zhou *et al.* 2020); A6-26, E4-63, E86-69, PG5068, PG6359, and PG0019 (Tang *et al.* 2022); Atlantic v.2.0, Castle Russet v.2.0, Avenger, Altus, Columba, and Spunta (Hoopes *et al.* 2022); Otava v.1 (Sun *et al.* 2022); and C88 v.1 (Bao *et al.* 2022). The genomic sequence of Soltu.DM.08G000640 (*StMSH4* in DM v.6.1) was used as query, and the results were manually curated to stitch together partial hits within 5 kb of each other, assumed to be truncated by structural variations. Incomplete hits matching with scaffold ends were removed from the analysis. Genic and 500 bp upstream and downstream regions from those genome assemblies, homologous to *Soltu.DM.08G000640*, were collected for haplotype analysis. The *StMSH4.f1* haplotype, associated with the CO shortage phenotype in our mapping population, was constructed in Geneious Prime 2022.2.2 (https://www.geneious.com) using the merged bam files the 102 true-positive CO shortage mutant clones.

### Multiple sequence comparison

Multiple sequence comparison of *StMSH4* alleles was performed in Geneious Prime 2022.2.2 using MUSCLE 3.8.425 (Edgar 2004) with default parameters. The dendrogram was generated using the neighbor-joining algorithm with default settings and a minimal support threshold based on bootstrap value of 80%.

### Kompetitive allele-specific PCR marker analysis

Leaf samples of complementation test populations CRH and ERH were sent to Bejo (Warmenhuizen, The Netherlands) for Kompetitive allele-specific PCR (KASP) marker analysis (LGC Genomics GmbH, Berlin, Germany) following manufacturer protocols. KASP markers distinguishing between putatively functional and nonfunctional *StMSH4* alleles were developed. Primers are listed in Supplementary Table 3. KASP assay results were visualized using SNPviewer (lgcgroup.com/products/genotyping-software/snpviewer) to confirm correct segregation and genotype calling.

### A *k*-mer-based exploration of *StMSH4* insertion alleles in commercial germplasm

A *k*-mer-based methodology was employed to examine the occurrence of *StMSH4* insertion alleles within the potato commercial tetraploid germplasm. Whole genome sequencing reads (150 bp paired-ends) obtained from 134 tetraploid potato varieties [manuscript in preparation, National Center for Biotechnology Information (NCBI) accession number PRJNA944441] were transformed into *k*-mers using KMC v.3.1.0 (Kokot *et al.* 2017) with default parameters and the “-k31” option. Additionally, 5 *k*-mer sets specific to different *StMSH4* alleles were generated by *k*-merizing sequences of 39 nucleotides that overlapped with the T/C polymorphism of the KASP marker MSH4_777, sequences of 60 nucleotides that spanned both insertion boundaries of the *StMSH4.t1/t2* transposon, as well as a sequence of 67 nucleotides centered around the 7 bp footprint of *StMSH4.f1*. These 5 *k*-mer sets were subsequently intersected with the *k*-mer sets derived from the 134 varieties. The *k*-mers that lacked specificity, being present in nearly all varieties, were excluded from the analysis. This curation process culminated in 5 definitive sets of *k*-mers tagging different *StMSH4* polymorphisms: ks_MSH4_777:C containing 9 *k*-mers specific to *MSH4_777:C* (the C allele of KASP marker MSH4_777), ks_MSH4_777:T containing 9 *k*-mers specific to *MSH4_777:T* (the T allele of KASP marker MSH4_777), ks_StMSH4.f1 containing 31 *k*-mers specific to *StMSH4.f1* footprint, ks_StMSH4.t1/t2_start containing 22 *k*-mers specific to *StMSH4.t1/t2* transposon start, and ks_StMSH4.t1/t2_end containing 23 *k*-mers specific to *StMSH4.t1/t2* transposon end. The presence or absence of these variant-specific *k*-mers within the varieties, in conjunction with their respective frequencies, was used to infer the distribution of distinct *StMSH4* alleles within the commercial germplasm (Supplementary Table 4). The dosage of the *MSH4_777:C* allele was calculated by dividing the *k*-mer frequencies of ks_MSH4_777:C by the summation of the *k*-mer frequencies of both *ks_MSH4_777:C* and *ks_MSH4_777:T*. Considering the distinct *k*-mer pattern observed in varieties Royal and Summer Delight, characterized by the presence of only 2 *k*-mers specific to the StMSH4.f1 footprint haplotype, we proceeded to extract short reads containing these 2 *k*-mers from these 2 varieties. This was performed utilizing the filter function of kmc_tools (Kokot *et al.* 2017). The resulting short reads were aligned to the potato reference genome DM v.6.1 using BWA-MEM algorithm v.0.7.17 (Li and Durbin 2009) with default parameters.

## Results

### CO shortage is controlled by a single locus on chromosome *8*

**Fig. 2.**
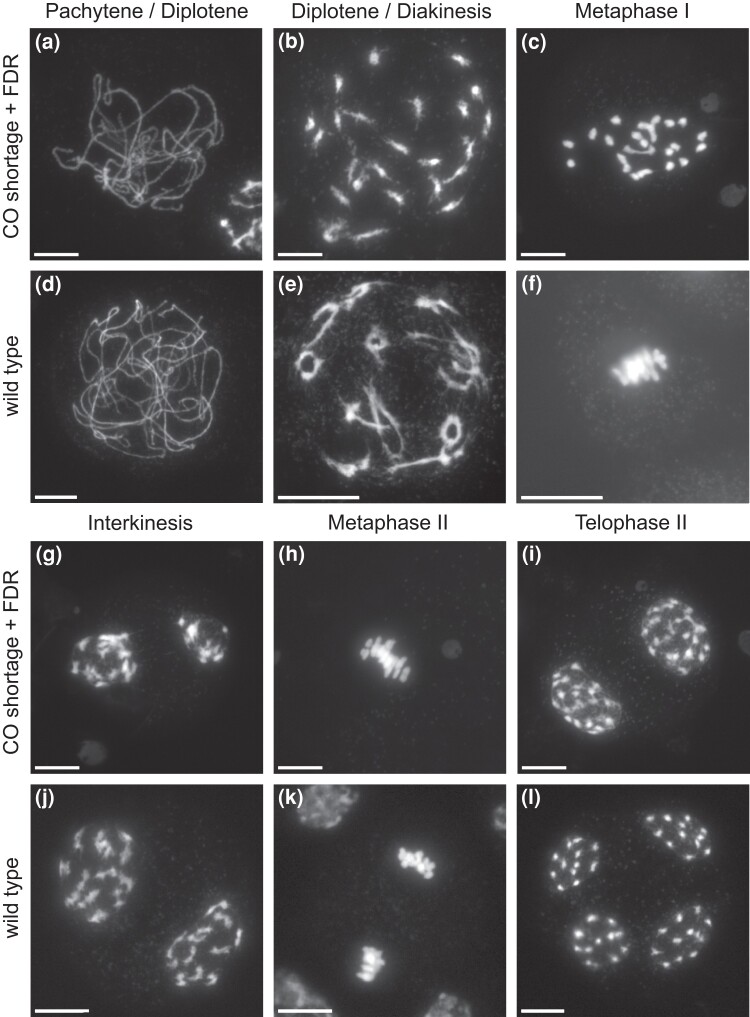
Cytological observation of male meiosis in clone CE-XW-1477 (a–c, g–i) classified as CO shortage mutant and clone RH89-036-16 (d–f, j–l) used as wild-type control. a, d) Pachytene/early diplotene. b, e) Late diplotene/early diakinesis. c, f) Metaphase I. g, j) Interkinesis. h, k) Metaphase II. i, l) Late telophase II with dyad (i) and (l) tetrad.

Stained pollen samples from 1,345 seedlings of the CE-XW population were observed under a microscope and classified into unknown, CO shortage mutant, and wild-type samples according to the dichotomous key presented in Fig. 1b. To confirm that our classification based on pollen microscopy corresponds to the expected cytological phenomenon of CO shortage and FDR, we observed male meiosis progression in clone CE-XW-1477, classified as CO shortage mutant, and in clone RH89-039-16 used as wild-type control. During pachytene, the 2 clones were indistinguishable, showing apparently normal chromosome pairing and synapsis (Fig. 2a). However, during late diplotene/early diakinesis, as the desynapsed chromosomes condensed, it became evident that, in clone CE-XW-1477, most homologs pairs lacked chiasmata, resulting in the presence of univalents during metaphase I (Fig. 2b and c). This marked a sharp departure from wild-type meiosis where ring and rod bivalents, indicative of chiasmata, are observed during late diplotene/early diakinesis (Fig. 2e). The presence univalents at metaphase I had consequential effects, leading to missegregation during the first meiotic division and to the presence of unbalanced products at interkinesis (Fig. 2g). However, during the second meiotic division, the fusion of metaphase II plates (Fig. 2h) effectively restituted the products of the first meiotic division and led to the formation of a balanced dyad (Fig. 2i). This contrasted with wild-type meiosis where 2 perpendicular metaphase II plates were observed, leading to the formation of a balanced tetrad (Fig. 2k and l). These observations confirmed the presence of CO shortage and FDR in clone CE-XW-1477 classified as CO shortage mutant via pollen microscopy.

Returning to pollen microscopy, our observations allowed classification of 134 individuals as CO shortage mutant and 912 as wild-type, while 299 offspring remained unclassified due to either low pollen stainability or uncertainty about pollen ploidy. To validate this classification, a subset of 500 individuals were regrown from tubers the following year, from which 470 pollen samples could be classified. In 107 mutant and 333 wild-type clones, we observed the same phenotype in both years. Five mutant clones were classified as wild-type in the 2nd year, and 25 wild-type clones were classified as mutant in the 2nd year. These 30 conflicting classifications (6.3%) were specifically related to individuals with low pollen shed and were discarded from further analysis. Ultimately, we mapped the *Ds-1* locus using 106 clones classified as CO shortage mutant and 857 classified as wild-type. While the classification of all mutant individuals was based on 2 years’ data, this was not the case of all wild-type individuals. Nonetheless, we decided to use all genotyped individuals classified as wild-type based on a single year observation, considering that the increase in power offered by a larger cohort will compensate for putative misclassification.

**Fig. 3.**
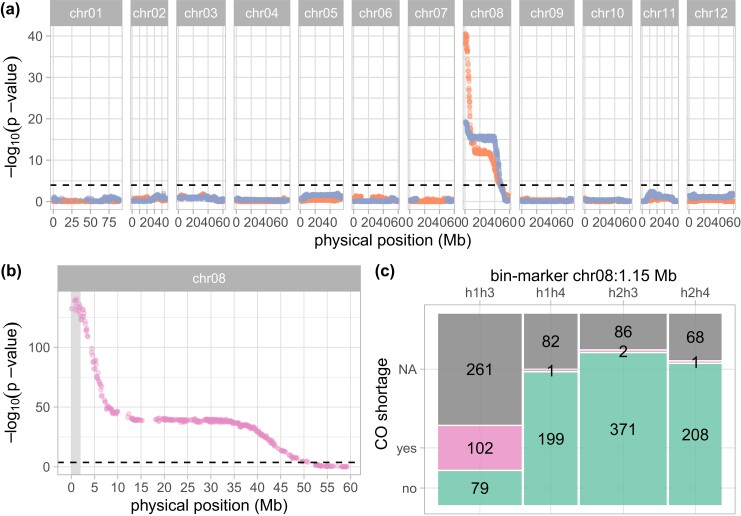
a, b) Significance of the association between markers and the phenotypic classification of CO shortage (*n* = 963). The X axis represents the physical position (Mb), the Y axis represents −log_10_(*P*-value), and the threshold of significance is indicated by the black dashed line. In a), clone C marker data are displayed in orange and clone E in blue, while in b), the integrated marker allele combination h1h3 is displayed in pink, and the candidate region for *Ds-1* is highlighted in gray. c) Mosaic plots illustrating the goodness of fit between phenotypic and genotypic observations for the markers at 1.15 Mb on chromosome *8*. Individuals phenotyped as wild-type or CO shortage mutant are displayed in green and pink, respectively, and phenotypically unclassified individuals in gray.

Using this binary phenotypic classification for QTL discovery, we mapped the *Ds-1* locus to the north arm of chromosome *8* at 1.15 Mb both on the female and the male physical maps. Merging parental markers of identical physical map positions, resulting in full classification, also localized *Ds-1* at 1.15 Mb (Fig. 3a and b). A recessive inheritance of CO shortage implies that the recessive phenotype will match only with one of the allele combinations h1h3, h1h4, h2h3, and h2h4 where h1 and h2 represent the 2 possible female haplotype and h3 and h4 the 2 possible male haplotypes. Indeed, at 1.15 Mn on chromosome *8*, 3 marker classes accurately predict wild-type plants where h1h4, h2h3, and h2h4 are associated with the *ds-1/Ds-1*, *Ds-1/ds-1*, and *Ds-1/Ds-1* genotypes, respectively, with only 1 or 2 phenotypic misclassifications, as shown in the mosaic plot (Fig. 3c). The allele combination h1h3 however, indicative of the *ds-1/ds-1* genotypes classified as CO shortage mutant, showed 102 true-positive and 79 false-negative classifications. These false-negatives could suggest a second 1:1 segregating locus compensating the *ds-1/ds-1* mutants. However, a new QTL analysis for CO shortage within the cohort of individuals with the allelic combination h1h3 at 1.15 Mb on chromosome *8* did not identify such a compensatory locus (Supplementary Fig. 2). Upon microscopic re-examination of the slides of 20 random false-positive clones, 5 are better classified as ambiguous due to a low pollen shed, 1 was confirmed wild-type, and 14 were reclassified as CO shortage mutant. Those 14 clones show a stainability above 50% with more than 95% of stained pollen grain being 2n. The occasional observation of a few small and stained pollen grains in those clones was in retrospect also visible in 10 randomly re-examined true-positive mutant clones. This suggests that mutant clones lacking COs can produce up to 3% of stained small pollen grains, assumed to be haploid or aneuploid cells with a stainable cytoplasm. Overall, false-positives were essentially explained by erroneous phenotypic classifications due to a too mild threshold to classify a pollen sample as wild-type based on a single stained small pollen grain or the potential misidentification of 2n pollen as n pollen in mutant clones lacking COs and producing elevated levels of 2n pollen production. Finally, after a recombinant analysis in the subset of individuals curated for false-positives and with unambiguous recombination breakpoints, we identified the first 1.9 Mb of chromosome *8* as candidate region for CO shortage (Supplementary Table 1).

### Candidate gene exploration

The identified candidate region encompasses a total of 129 genes, of which 14 have been annotated as being involved in either reproduction (GO:0000003) or cell cycle (GO:0007049). Within this subset, a single meiotic gene, *Soltu.DM.08G000640*, was identified. This gene appears to be the ortholog of *AtMSH4* and is henceforth denoted as *StMSH4*. In *A. thaliana*, *Atmsh4* mutants exhibit a severe reduction in fertility due to an 85% reduction in chiasmata frequency at metaphase I leading to univalents and unbalanced chromosomal segregation (Higgins *et al.* 2004). This phenotype, typical of mutants in the ZMM pathway, strikingly resembles the meiotic observations made in our mapping population (Fig. 2; Jongedijk and Ramanna 1989). To pinpoint potential causal mutations, we performed variant calling on the bulked skim-sequencing data of the 102 true-positive mutant clones lacking COs. We identified a total of 26,327 high-confidence variants in the candidate region and annotated them using SnpEff. Through subsequent filtering with SnpSift, we isolated 24 LOF mutations. Within this subset, 6 variants were homozygous for the alternative (null) allele in our bulk (Supplementary Table 2). These 6 variants were distributed across 4 different genes: *Soltu.DM.08G000140* encoding for a YTH family protein, *Soltu.DM.08G000470* encoding for a hAT transposon superfamily, *Soltu.DM.08G000800* encoding for an NB-ARC domain-containing disease resistance protein, and *Soltu.DM.08G000640* (*StMSH4*) encoding for a MutS-like protein 4. Our CO shortage bulk was fixed for a 7 bp insertion in *StMSH4* second exon which was predicted to lead to a frame-shift and a subsequent loss of function. Drawing from both cytological and genetic evidence, StMSH4 emerges as the most compelling candidate gene for the *Ds-1* locus.

### Natural diversity of *StMSH4* haplotypes in potato germplasm

**Fig. 4.**
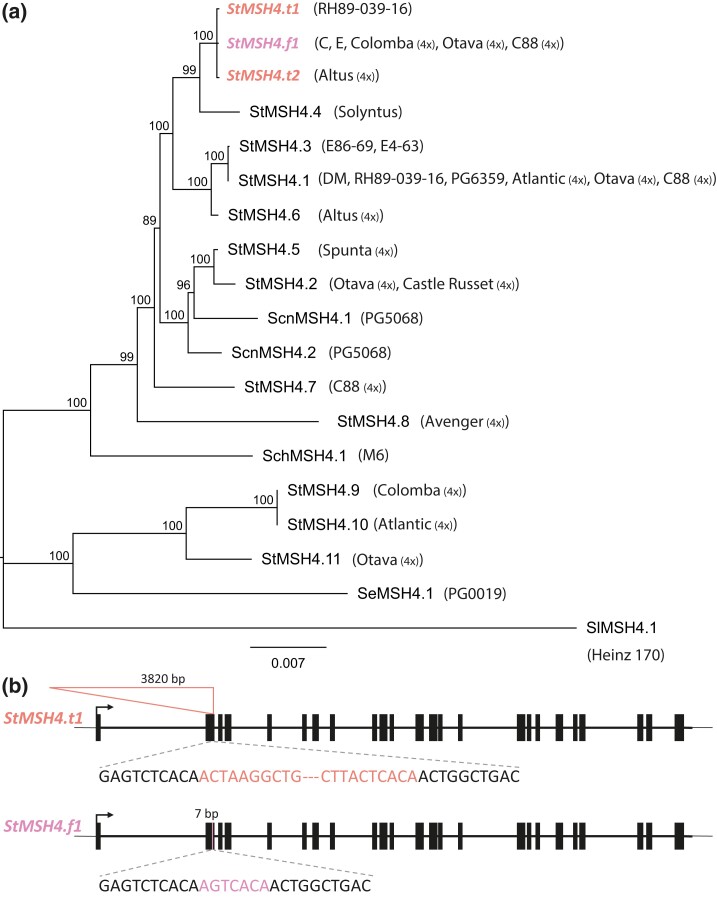
a) Dendrogram of *StMSH4* haplotypes retrieved from de novo assemblies. Bootstrap values are indicated at nodes. Clones in which the various haplotypes were identified are indicated in between brackets. Names of tetraploid clones are followed by (4×). Haplotypes with insertions in the second exon of *StMSH4* are indicated in pink and orange, respectively. b) Haplotypes *StMSH4.t1* and *StMSH4.f1* show insertions of 3820 and 7 bp in the second exon of candidate gene *StMSH4*.

Sequence data of *StMSH4* were retrieved with BLAST from 18 de novo–assembled genomes. This resulted in the identification of at least 19 unique haplotypes. We aligned these sequences with the haplotype of CE-XW individuals classified as CO shortage mutant and calculated a neighbor-joining tree of the haplotypes and rooted the tree using the haplotype from *S. lycopersicum* as outgroup (Fig. 4a). The most common haplotype, *StMSH4.1*, was identified in 6 different clones: DM, RH, Atlantic, PG6359, Otava, and C88. The mutant haplotype associated with CO shortage in our mapping population was identical to the second most common haplotype, named *StMSH4.f1* where the f indicates a footprint. This footprint haplotype was found in Colomba, Otava, and C88 and is characterized by a 7 bp insertion within the second exon of *StMSH4* as annotated in DM v.6.1. Strikingly, much longer insertions of 3,820 and 3,819 bp are observed at the exact same position in haplotypes named StMSH4.t1 and StMSH4.t2, where the t indicates a transposon insertion (Fig. 4b). These 2 haplotypes were found in clones RH89-039-16 and Spunta, respectively, and are 99.9% identical with only 10 SNPs and 1 indel observed within the insertion. Albeit the different insertions observed in haplotypes *StMSH4.t1*, *StMSH4.t2*, and *StMSH4.f1*, the remainder of the haplotypes are identical to each other (Supplementary File 1). We submitted the *StMSH4.t1* insert sequence to BLASTn against the RepetDB database (Amselem *et al.* 2019) “Solanum_tuberosum consensus from ReptDB v2” with default parameters. The best hit, with 93% sequence identity, returned the consensus sequence Stub_TedenovoGr-B-G5183-Map16. This sequence is classified as a Class II TIR transposon and is present in 294 copies and 342 fragments in DM v.4.03 and amounts for a cumulative genome coverage of 335,463 bp. In conclusion, we identified *StMSH4* as a candidate gene, and we assume that haplotypes with a premature stop codon due to a transposon insertion or footprint are associated with CO shortage.

### Complementation test

**Fig. 5.**
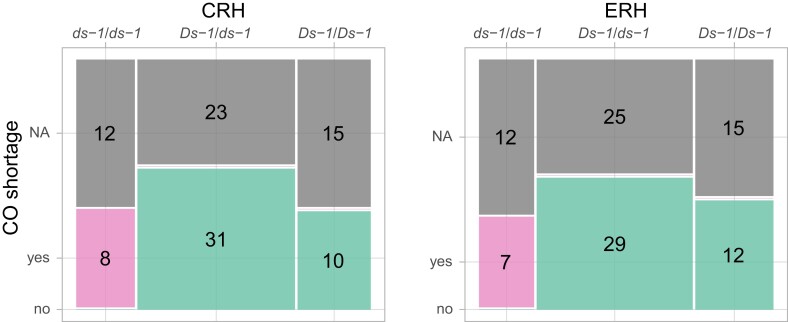
Mosaic plots illustrating the agreement between CO shortage phenotype and genotypes obtained with KASP markers tagging StMSH4 haplotypes in 2 complementation studies. CRH and ERH indicate the offspring from a cross between clone C or E with clone RH89-039-16. *Ds-1* indicates *StMSH4* alleles with no insertion, and *ds-1* stands for either *StMSH4.f1* or *StMSH4.t1*. Individuals phenotyped as wild-type or CO shortage mutant are displayed in green and pink, respectively, and phenotypically unclassified individuals in gray.

The evidence that the *Ds-1* locus is equal to *StMSH4* is only based on a positional colocalization and phenotypical similarity with *Atmsh4* mutants. To validate our positional evidence in another genetic background, we designed a genetic complementation assay using different insertion mutant versions of *StMSH4* as found in different potato clones. We crossed clones C and E, both heterozygous for *StMSH4.f1*, with clone RH89-039-16, heterozygous for *StMSH4.t1*, generating populations CRH and ERH. KASP marker MSH4_777 and MSH4_168, both tagging *StMSH4.t1* and *StMSH4.f1*, were used to follow the segregation of either haplotype in the progenies of CRH and ERH. As expected, in both populations, the KASP markers segregated in a 1:2:1 fashion and about one-quarter of offspring, homozygous for the KASP alleles associated with the insertion alleles, are assumed to carry the haplotype combination *StMSH4.t1*/*StMSH4.f1*. We phenotyped population CRH and ERH for CO shortage without prior knowledge on the seedling genotypes. Out of CRH 99 seedlings, 47 plants did not flower or shed sufficient pollen for microscopic observation and 3 plants displayed insufficient pollen stainability. Among the remaining 49 plants, 8 were classified as CO shortage mutant and 41 as wild-type. Similarly, for the 100 seedlings of ERH, 49 plants did not flower or shed sufficient pollen for microscopic observation and 3 plants displayed insufficient pollen stainability. Among the remaining 48 plants, 7 plants were classified as CO shortage mutant and 41 plants as wild-type. We observed a perfect correlation between our CO shortage classification and the 2 KASP markers distinguishing *StMSH4.t1* and *StMSH4.f1* from the functional haplotypes (Fig. 5). All phenotyped plants bearing the haplotype combination *StMSH4.t1*/*StMSH4.f1* were classified as CO shortage mutant. Although our population size does not allow us to rule out the presence of another mutation closely linked to *StMSH4*, these results strongly suggest that *StMSH4.t1* and *StMSH4.f1* alleles cannot complement each other and are nonfunctional. From now on, those nonfunctional alleles can be referred to under the unified notation *Stmsh4*.

### Occurrence of *StMSH4* insertion mutants in commercial tetraploid varieties

To assess the occurrence of *Stmsh4* alleles in commercial germplasm, we intersected the *k*-mer sets of 134 resequenced tetraploid varieties with the *k*-mer sets uniquely tagging the KASP marker allele *MSH4_777:C*, the footprint region of *StMSH4.f1* and the junctions at both ends of *StMSH4.t1* transposon insertion site (Supplementary Table 4). Surprisingly, we observed at least one of the mutant haplotypes *StMSH4.t1* or *StMSH4.t2* in 70 out of the 134 varieties, because in these varieties all *k-*mers unique to the junctions of both insertion ends were present. Furthermore, all footprint-specific *k*-mers were identified in 21 varieties including 14 varieties already positive for the transposon insertion. However in 2 other varieties, Royal and Summer Delight, only a subset of 2 footprint-specific *k*-mers were detected. Upon retrieving short reads containing these unique k-mers from these varieties and aligning them to the DM v.6.1 reference genome, we identified 2 new *StMSH4* haplotypes. Firstly, Royal's *StMSH4* haplotype, denotated as *StMSH4.f2*, was characterized by a 4 bp footprint located at the position and replacing the 7 bp insertion found in *StMSH4.f1* (Supplementary Fig. 3a). Secondly, Summer Delight retrieved reads mapped partially to the footprint location and partially to another region 28,460 bp downstream. This suggests that the transposon excision event caused a structural rearrangement producing another more complex footprint haplotype (*StMSH4.f3*) (Supplementary Fig. 3b). Whether the *StMSH4.f2* and *StMSH4.f3* alleles are also nonfunction remains to be demonstrated. Finally, we observed a perfect correlation between the 79 varieties positive for *k*-mers tagging *MSH4_777:C* and varieties positive for either *StMSH4* insertion mutants. This observation confirms that our KASP marker MSH4_777 accurately predicts the insertion alleles and can be used to estimate their combined dosage. We estimated that a total of 55 varieties were nulliplex for *MSH4_777:C*, while 45 were simplex, 26 were duplex ,and 8 were triplex. Hence, with a minor allele frequency (MAF) estimated at 22.6%, deleterious *StMSH4* insertion mutants are commonly present in commercial germplasm.

## Discussion

### Phenotypic classification of CO shortage via pollen microscopy

Rather than relying on laborious cytological observations of male meiocytes to phenotype CO shortage, we exploited the combination of CO shortage and FDR 2n pollen in our population to phenotype this trait through pollen microscopy. Either method has its advantages, where meiocyte observations would allow to capture the reduced chromosome pairing, while pollen observations allow a quick and dirty classification of a large population. A disadvantage of this latter approach is that CO shortage could only be positively classified and distinguished from other causes of male sterility, when combined with FDR 2n pollen production. This requirement for 2n pollen resulted in 261 unclassified ds*-1*/*ds-1* individuals (59.0%), compared with 236 unclassified *Ds-1*/— individuals (23.2%). Classification of wild-type offspring was highly accurate with only 4 misclassifications (0.5%), allowing a high mapping accuracy despite 79 misclassifications (43.6%) observed for descendants classified as CO shortage mutant. We learned that those misclassifications were predominantly caused by 2 misleading phenotypic observations: (1) almost all stainable pollen grains were unreduced, and we lacked small-sized pollen grains to see that, and (2) we observed a few stainable small pollen grains, while this possibility was not expected in clones lacking COs (initial threshold 0%). Stainability of small pollen grains in individuals with CO shortage suggests that balanced segregation may occur by chance alone or that despite an unbalanced number of chromosome the cytoplasm of pollen grains may still be stainable. This led to a new threshold (<5%), which was successfully applied during the phenotypic classification of the complementation test populations.

### Different *StMSH4* insertion alleles

In this study, we identified a variety of *StMSH4* alleles, including nonfunctional alleles due to a transposon insertion in the second exon of this gene or to a 7 bp footprint at a position coinciding with the transposon insertion site. Among them, the transposon insertion alleles *StMSH4.t1* and *StMSH4.t2*, indistinguishable based on our *k*-mer-based analysis, were the most widespread (Supplementary Table 4). The low sequence divergence between *StMSH4.t1* and *StMSH4.t2*, limited to 10 SNPs and a 1 bp indel within the insertion sequence, can be explained in 3 ways. Either those 2 alleles are *bona fide* and result from (1) independent transposition events at the same location in the same ancestral allele (very unlikely), or (2) from a single transposon insertion event and subsequent divergency restrained to the transposon sequence, or (3) these differences are artifacts caused by the challenge of assembling repetitive sequences present in high copy number in the genome. *StMSH4.f1*, the 7 bp footprint allele causing CO shortage in our mapping population, was also relatively common among tetraploid varieties. However, we could also identify 2 rarer footprint alleles (Supplementary Fig. 3) and cannot exclude the future discovery of more footprints resulting from independent excision events of the widespread transposon alleles. Finally, the prevalence of *Stmsh4* nonfunctional alleles within the cultivated germplasm of potato agrees with earlier report of tetraploid variety Chippewa, a major contributing ancestor to the North American germplasm (Love 1999), being duplex for the recessive *ds-1* allele (Matsubayashi 1979; Jongedijk and Ramanna 1988).

### *MSH4* and meiotic adaptation to polyploidy

In allotetraploid *Brassica napus*, a reduction in the number of functional copies of *MSH4* to a single copy prevents COs between homeologous chromosomes without affecting the total number of COs (Gonzalo *et al.* 2019). In this context, reducing *MSH4* dosage seems to help stabilizing allopolyploid meiosis by favoring homologous chromosomes as recombination partner. Contrary to allopolyploids, the stabilization of meiosis in autopolyploids, such as *S. tuberosum*, does not rely on recombination partner choice but on avoiding multivalent formation, in particular the combination of trivalent and univalent (Bomblies 2022). This can be mediated by increasing CO interference strength and ultimately decreasing CO number to a minimum of 1 per pair (Morgan *et al.* 2021). Interestingly, recent cytological investigation of tetraploid potato cultivars revealed that clone Sante display significantly less multivalent than clones Maris Peers and Cara (Choudhary *et al.* 2020). Remarkably, Maris Peer and Cara are quadruplex for functional *StMSH4* alleles (Supplementary Table 4) and Sante appears to bear 1 copy of *StMSH4.t1* [S. Oome (HZPC), personal communication]. Whether the prevalence of *Stmsh4* mutant alleles in tetraploid potatoes is simply due to relaxed purifying selection or if it can be explained by an adaptation to polyploidy (i.e. minimizing multivalents in autotetraploid *S. tuberosum* or homeologous COs in allotetraploid wild relatives such as *Solanum stoloniferum*) could be investigated with functional experiments.

### CO shortage: a double-edged sword for breeders

At a first glance, the production of sterile n gametes due to CO shortage appears to be a detrimental phenotype that should be removed from potato breeding programs. Suboptimal fertility has always been an issue for potato breeders (Krantz 1924) and maintaining fertility upon inbreeding is central to the reinvention of potato as a diploid inbred line-based crop. With a MAF estimated at 22.6% in tetraploid varieties, *Stmsh4* alleles are contributing to the notorious fertility problems of dihaploids induced from cultivars. Ignoring double reduction, the probability to induce a dihaploid with a given number of *Stmsh4* copies can be calculated using Equation (1) with *p* being the ploidy of the parent *P*, $P(P_{n})$ being the probability of parent *P* to bear *n* of copies of *Stmsh4*, and $P(G_{k})$ being the probability of a gamete *G* (future dihaploid) to bear *k* copies of *Stmsh4*.


(1) $$P(G_{k})=\sum_{n=0}^{p}P(P_{n})\times \frac{C_{k}^{n}\times C_{(p/2)-k}^{p-n}}{C_{p/2}^{p}}.$$

Extrapolating from the estimated dosages of *Stmsh4* in commercial varieties (Supplementary Table 4), we can estimate that 6.4% of all induced dihaploids will be homozygous for *Stmsh4* and 33.5% of them will be heterozygous. While these dihaploids are often used to introduce quality and resistance traits to the diploid gene pool, they will also introduce *Stmsh4* alleles, thus hampering future inbreeding efforts. Indeed, one-quarter of the S1 population obtained by selfing a clone heterozygous for *Stmsh4* will display CO shortage. CO shortage will only be observed and selected against when attempting to produce an S2, hereby wasting labor and greenhouse space. We therefore recommend to breeders that aim at developing fertile diploid inbred lines to remove *StMSH4.f1* and *StMSH4.t1*/*t2* haplotypes from their germplasm.

Looking at CO shortage from another angle, one could envisage to combine it with another meiotic mutation, FDR 2nG, and exploit these highly heterozygous and highly uniform unreduced gametes to create tetraploid varieties via sexual polyploidization. Interploidy breeding schemes exploiting the simplicity of diploid breeding and the heterozygosity offered by tetraploids have long been proposed in potato (Chase 1963; Hutten 1994). In those schemes, tetraploid varieties are produced by unilateral (4x × 2x) or bilateral (2x × 2x) sexual polyploidization with diploid clones producing 2nG. However, not all 2nG are made equal with FDR 2nG retaining ∼80% of parental heterozygosity compared with ∼40% for 2nG formed by a second division restitution (SDR) (Douches and Quiros 1988a, 1988b; Jongedijk, Hutten, *et al.* 1991; Peloquin *et al.* 2008). This higher heterozygosity of FDR 2nG has been linked with a significant yield increase in progenies when compared with SDR 2nG (Kidane-Mariam and Peloquin 1975; Mendiburu and Peloquin 1977; Hutten *et al.* 1994). In clones with CO shortage, the significant reduction in CO number boosts the heterozygosity of FDR 2nG to 94.1% (Jongedijk, Hutten, *et al.* 1991) and, thus, could contribute to an even higher heterosis in the tetraploid progeny. Moreover, the increased uniformity of these 2nG will also be instrumental to the development of relatively uniform tetraploid varieties grown from true seeds (TPS varieties), without the necessity to develop fully inbred parents. Practically, breeders can generate diploid progenitor material which are heterozygous for *Stmsh4*. These progenitor clones can be used as recurrent parent at the diploid level, but after 1 generation of selfing, the descendants with CO shortage and FDR 2nG could be used for commercial true seed production. Likewise, descendants with CO shortage can also be obtained in hybrid progeny descending from a cross between diploid parents that both carry an *Stmsh4* allele. Nonetheless, it is important to keep in mind that the presence of heterozygous regions between residual COs and the ends of chromosomes would compromise the uniformity of the resulting progeny.

Despite being less common than FDR 2n pollen production, the formation of FDR 2n megaspores has been reported in potato clones with CO shortage (Jongedijk, Ramanna, *et al.* 1991). Those authors also discussed the potential use of such mutants to produce heterozygous and relatively uniform tetraploid TPS varieties via bilateral sexual polyploidization. While being facilitated by the potential of marker-assisted selection for CO shortage, further research on the genetic regulation of FDR 2nG production both on the male and the female sides remains essential to efficiently exploit CO shortage. Importantly, the genetic regulation of FDR 2nG production is expected to be more complex on the female than on the male side because of the successive type of cytokinesis of female meiosis. FDR 2n pollen could be achieved with a single mutation as in the cases of *Atps1* and *Atjason* mutants (d’Erfurth *et al.* 2008; De Storme and Geelen 2011). Conversely, obtaining near nonrecombinant unreduced female gametes is likely to necessitate the interplay of several mutations. Such mutations could lead to either the abolition of cytokinesis following meiosis I coupled with the parallel orientation of spindles during meiosis II or to the loss of cohesion between sister chromatids during meiosis I coupled with the omission of the second meiotic division.

## Supplementary Material

iyad194_Supplementary_Data

## Data Availability

The sequencing data of population CE-XW are available from the European Nucleotide Archive (ENA) under the BioProject ID PRJEB56778. The alignment and the raw fasta files of *MSH4* haplotypes, the phenotyping data, genotyping data, and all the R codes necessary to reproduce the results and figures of this article are available at https://doi.org/10.6084/m9.figshare.22047965.v1. Diploid potato (USW5337.3), E (77.2102.37), and RH89-039-16 are available from the Department of Plant Breeding at Wageningen University & Research upon request. Clones C (USW5337.3) and RH89-039-16 are also available from the Potato Introduction Station, Sturgeon Bay, Wisconsin, under the accession names GS 218 and PI 675351, respectively. Supplemental material available at GENETICS online.
