# Supplementary material for: Crossover shortage in potato is caused by StMSH4 mutant alleles and leads to either highly uniform unreduced pollen or sterility
Source: Genetics. 2023 Nov 7;226(1):iyad194. doi: 10.1093/genetics/iyad194 (PMC10763545; doi:10.1093/genetics/iyad194)
Supplement: iyad194_Supplementary_Data [file iyad194_supplementary_data.zip › Figure_S1_GENETICS-2023-306474.pdf]

Figure S1

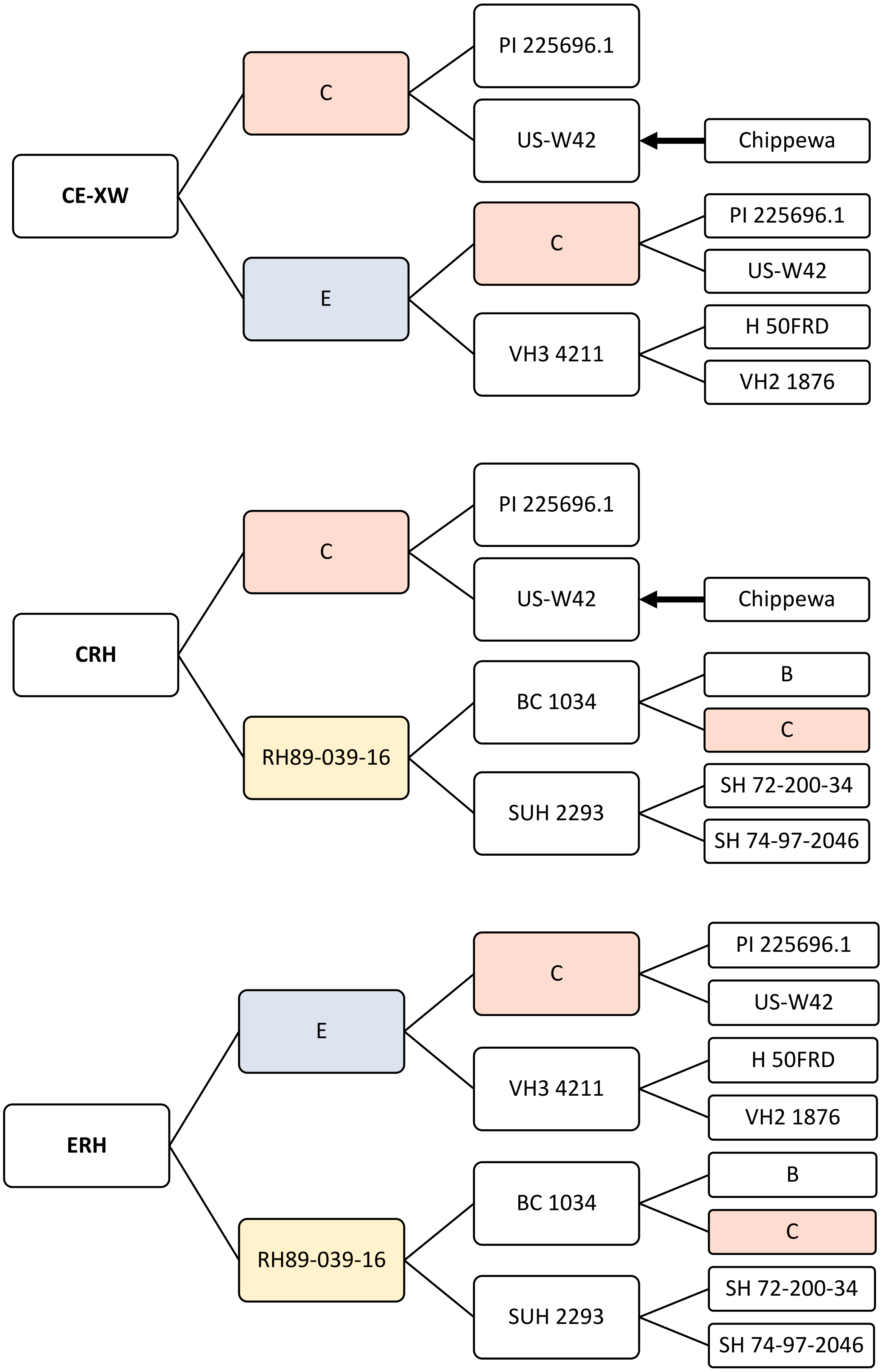

Pedigrees of bi-parental diploid potato populations CE-XW, CRH and ERH. Parental clones C, E and RH89-039-16 are highlighted in orange, purple and yellow respectively. Bold arrows represent dihaploidisation.
