# Supplementary material for: Crossover shortage in potato is caused by StMSH4 mutant alleles and leads to either highly uniform unreduced pollen or sterility
Source: Genetics. 2023 Nov 7;226(1):iyad194. doi: 10.1093/genetics/iyad194 (PMC10763545; doi:10.1093/genetics/iyad194)
Supplement: iyad194_Supplementary_Data [file iyad194_supplementary_data.zip › Figure_S2_GENETICS-2023-306474.pdf]

Figure S2

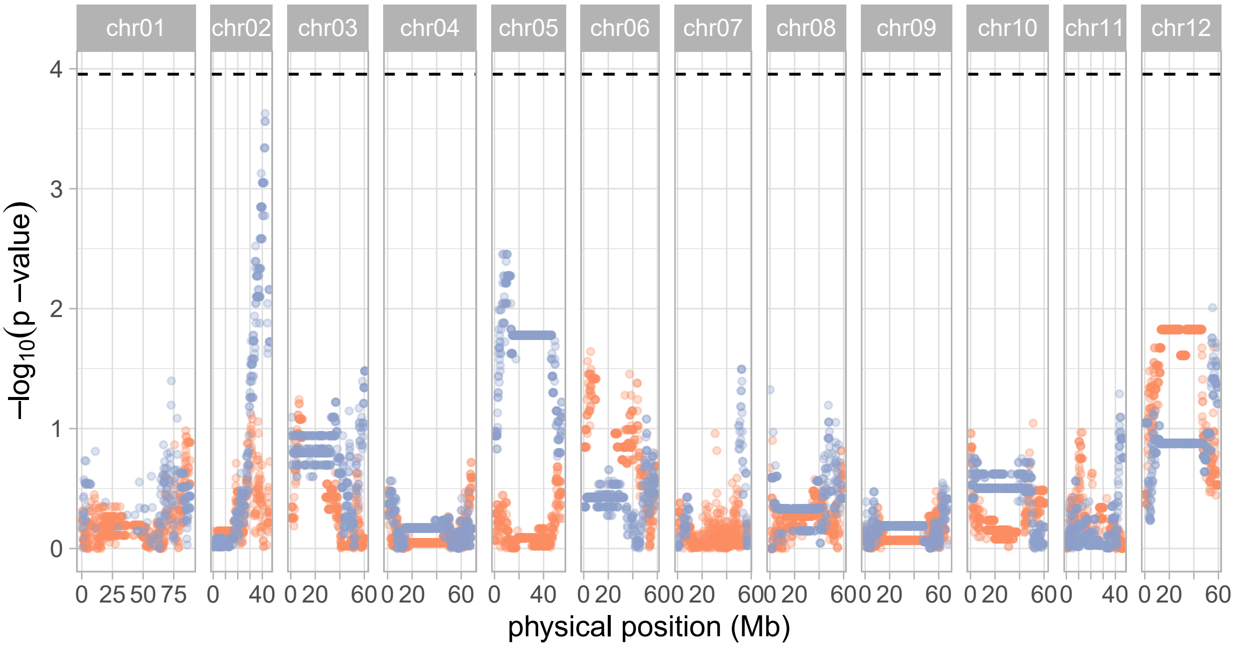

Significance of the association between parental markers and the phenotypic classification of CO shortage within the cohort of individuals with the allelic combination h1h3 at 1.15 Mb on chromosome *8* (n=181). The X axis represents the physical position (Mb), the Y axis represents -log10(*p*-value) and, the threshold of significance is indicated by the black dashed line.
