# Supplementary material for: Crossover shortage in potato is caused by StMSH4 mutant alleles and leads to either highly uniform unreduced pollen or sterility
Source: Genetics. 2023 Nov 7;226(1):iyad194. doi: 10.1093/genetics/iyad194 (PMC10763545; doi:10.1093/genetics/iyad194)
Supplement: iyad194_Supplementary_Data [file iyad194_supplementary_data.zip › Figure_S3_GENETICS-2023-306474.pdf]

Figure S3

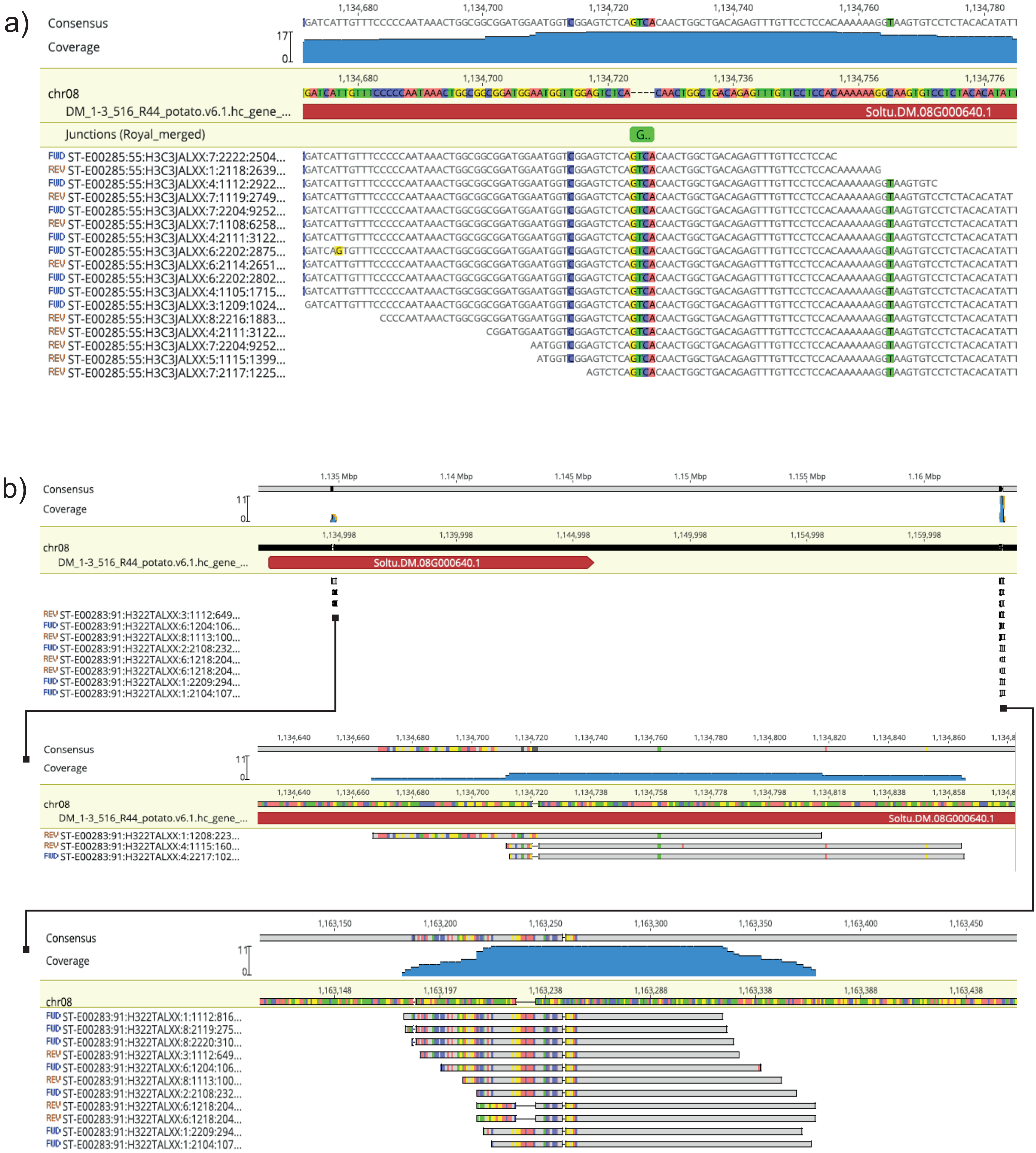

Alignment to DM v.6.1 of short-reads containing footprint specific *k*-mers extracted from a) Royal showing a 4 bp footprint and from b) Summer Delight showing similarity with both the transposon insertion site in and another region ~28 kbp downstream.
